# Interplay between Non-Coding RNA Transcription, Stringent/Relaxed Phenotype and Antibiotic Production in *Streptomyces ambofaciens*

**DOI:** 10.3390/antibiotics10080947

**Published:** 2021-08-05

**Authors:** Eva Pinatel, Matteo Calcagnile, Adelfia Talà, Fabrizio Damiano, Luisa Siculella, Clelia Peano, Giuseppe Egidio De Benedetto, Antonio Pennetta, Gianluca De Bellis, Pietro Alifano

**Affiliations:** 1 Institute of Biomedical Technologies, National Research Council, 20090 Segrate, Italy; eva.pinatel@itb.cnr.it (E.P.); gianluca.debellis@itb.cnr.it (G.D.B.); 2 Department of Biological and Environmental Sciences and Technologies, University of Salento, Via Monteroni, 73100 Lecce, Italy; matteo.calcagnile@unisalento.it (M.C.); adelfia.tala@unisalento.it (A.T.); fabrizio.damiano@unisalento.it (F.D.); luisa.siculella@unisalento.it (L.S.); 3 Institute for Genetic and Biomedical Research, Operative Unit (UoS) of Milan, National Research Council, 20089 Rozzano, Italy; clelia.peano@irgb.cnr.it; 4 Human Technopole, 20157 Milan, Italy; 5 Department of Cultural Heritage, University of Salento, Via Monteroni, 73100 Lecce, Italy; giuseppe.debenedetto@unisalento.it (G.E.D.B.); antonio.pennetta@unisalento.it (A.P.)

**Keywords:** re-defined transcriptome, ncRNA, *Streptomyces*, stringent response, antibiotic production

## Abstract

While in recent years the key role of non-coding RNAs (ncRNAs) in the regulation of gene expression has become increasingly evident, their interaction with the global regulatory circuits is still obscure. Here we analyzed the structure and organization of the transcriptome of *Streptomyces ambofaciens*, the producer of spiramycin. We identified ncRNAs including 45 small-RNAs (sRNAs) and 119 antisense-RNAs (asRNAs I) that appear transcribed from dedicated promoters. Some sRNAs and asRNAs are unprecedented in *Streptomyces* and were predicted to target mRNAs encoding proteins involved in transcription, translation, ribosomal structure and biogenesis, and regulation of morphological and biochemical differentiation. We then compared ncRNA expression in three strains: (i) the wild-type strain; (ii) an isogenic *pirA*-defective mutant with central carbon metabolism imbalance, “relaxed” phenotype, and repression of antibiotic production; and (iii) a *pirA*-derivative strain harboring a “stringent” RNA polymerase that suppresses *pirA*-associated phenotypes. Data indicated that the expression of most ncRNAs was correlated to the stringent/relaxed phenotype suggesting novel effector mechanisms of the stringent response.

## 1. Introduction

In recent years, the key role that small non-coding RNAs (ncRNAs) play in the regulation of gene expression in bacteria is becoming increasingly evident. ncRNA-mediated gene regulation involving riboswitches, *trans*-encoded small RNAs (herein referred to as sRNAs), or *cis*-encoded antisense RNAs (herein referred to as asRNAs) modulates many essential physiological and stress responses [1,2,3,4]. Among ncRNAs, sRNAs and asRNAs mostly act on target mRNAs via base-pairing leading to positive or negative modulation of the target gene expression, providing the bacterial cell with a very simple, cheap and effective gene regulation mechanism that is alternative to more complex and expensive mechanisms based on protein-nucleic acid interactions [5,6].

sRNAs are encoded elsewhere in the genome with respect to gene coding for their target mRNAs and are usually able to act on multiple targets although with a limited base pairing that often requires RNA chaperone proteins [5,6,7]. asRNAs and their target mRNAs are, instead, produced by overlapping transcription, and are thus characterized by extensive and perfect base pairing. Essentially, two different mechanisms can generate asRNAs in bacteria: (i) promoter(s) within coding or non-coding genes, including their 5′-UTR, oriented in the divergent direction; and (ii) a read-through of the transcriptional terminator(s) of a contiguous gene that is transcribed in convergent direction, due to either leaky termination or specific anti-termination mechanisms.

Biological effects of asRNAs may vary depending on their relative expression and the genetic and physical context in which they are found. On one hand, there are many studies that contend that a large part of asRNAs may be considered as transcriptional noise arising from either spurious promoters or leaky terminators, and without physiological significance [8,9,10,11,12]. Indeed, there is evidence that the number of asRNAs is exponentially dependent on the genomic AT content, and that expression of asRNAs at low levels exerts little impact in terms of energy consumption [13]. On the other hand, there are examples of asRNAs that modulate gene expression both by either transcriptional or post-transcriptional mechanisms [7,14]. The first ones involve either modulation of transcription termination or transcription interference due to promoter occlusion, RNA polymerase collision or sitting-duck interference; the second ones involve either enhancing or decreasing either target mRNA stability, translation, or both [7,14]. Therefore, one must be very cautious before excluding the biological relevance of a particular asRNA, especially if the asRNA was preserved during evolution.

Small regulatory RNAs were initially discovered and studied in *Escherichia coli* [15,16]. The advent of high-throughput sequencing technologies has spurred the systematic investigation of ncRNAs in a wide range of bacteria [17], and their importance has been demonstrated in a multitude of bacterial adaptive processes including quorum sensing, biofilm formation, and virulence [18,19]. ncRNAs have more recently been explored in streptomycetes [20,21,22,23], soil bacteria relevant for their mycelial morphology and cell cycle, their ability to produce a wide array of secondary metabolites of industrial interest, and complex gene regulation networks [24]. There is evidence that these bacteria ncRNAs play a role in managing the regulation of primary metabolism, stress responses, morphological differentiation, and secondary metabolism [25,26,27,28]. Elucidating ncRNA regulators and their corresponding networks will provide us with the opportunity to powerfully engineer the metabolism of these industrially relevant microorganisms [29].

Among streptomycetes, *Streptomyces ambofaciens* ATCC 23877 has been known for the last 65 years due to its capability of producing a wide array of bioactive compounds including spiramycin (a macrolide used in human medicine as an antibacterial and antiparasitic agent) [30,31], congocidine (pyrrolamide with a broad range of biological activities but no medical application) [32], and many other compounds that were revealed by genome mining-guided approach [33]. In the current study, we applied RNAseq technology to analyze differential expression of ncRNAs in three *S. ambofaciens* strains: the wild type ATCC 23877 strain, an isogenic *pirA*-defective mutant (ΩpirA) that is characterized by central carbon and energy metabolism imbalance, high sensitivity to oxidative injury, relaxed phenotype, and repression of polyketide antibiotic production [34,35], and derivative strain harboring a stringent RNA polymerase exhibiting partial suppression of *pirA*-defective phenotypes. The ΩpirA mutant was generated by site-specific integration of plasmid pTYM-18 (a ΦC31 Att/Int system-based vector) that inactivates *pirA*, encoding an evolutionarily conserved in *Streptomyces* redox-sensitive negative modulator of very-long-chain acyl-CoA dehydrogenase AbdB, which catalyzes the first committed step of the beta-oxidation pathway [35]. The direct effect of AcdB inhibition is an imbalance of the acyl-CoA pool that contains fundamental precursors for the biosynthesis of antibiotics and other secondary metabolites [35]. In addition to carbon and energy metabolism imbalance the ΩpirA strain exhibits a high sensitivity to oxidative injury, suggesting a role of PirA in the redox state control [34,35], and a relaxed phenotype with marked downregulation of genes coding for ribosomal proteins and translation factors, and upregulation of genes involved in amino acid biosynthesis [35]. The initial aim of the present study was to characterize the ΩpirA mutant in more detail, focusing on some aspects related to its relaxed phenotype including ncRNA expression. To this purpose we introduced into this strain the *Nonomuraea gerenzanensis rpoB(R)* gene, coding for a mutant type of RNA polymerase beta-chain [36]. The product of *rpoB(R)* is characterized by five amino acid substitutions located within or close to the so-called rifampin resistance clusters that play a key role in mimicking a “stringent” phenotype [36,37], by activating the secondary metabolism. Indeed, *rpoB(R)* markedly activated antibiotic biosynthesis in the wild-type *Streptomyces lividans* strain 1326 and also in strain KO-421, a relaxed (*rel*) mutant unable to produce ppGpp [36]. The rationale of transcriptomic approach of the present study was to better understand the interplay between ncRNA expression and control of morphological and biochemical differentiation.

## 2. Results

### 2.1. S. ambofaciens Transcriptome: Structure and Organization

The transcriptome of *S. ambofaciens* ATCC 23877 linear chromosome was redefined from RNAseq data of bacteria growing in YS broth at different time points (48 h, 72 h, 96 h, and 120 h) based on Rockhopper software [38,39,40] predictions. The original 7229 genes located on the linear chromosome were structured in 5587 transcriptional units (TUs). Furthermore, 4433 TUs were monocistronic while 1154 were polycistronic, with a maximum of 23 genes included in a single unit (Table 1, and Supplementary Table S1). Only 53% of the originally defined genes (2944) retained the exactly original annotation while, for the other genes, either new 5′-UTR, 3′-UTR, or both, sequences, or polycistronic arrangements were individuated. The new annotation increased the portion of linear chromosome nucleotides included in the transcriptome from 87% to 90.8%. This 3.8% increase leads to a doubling of the mapped reads falling into annotated transcripts, which increase from 45% to 94% on average on *S. ambofaciens* ATCC 23877 strain. This gain is even more impressive looking at the 48 h timepoint (Supplementary Table S2).

**Figure 1:**
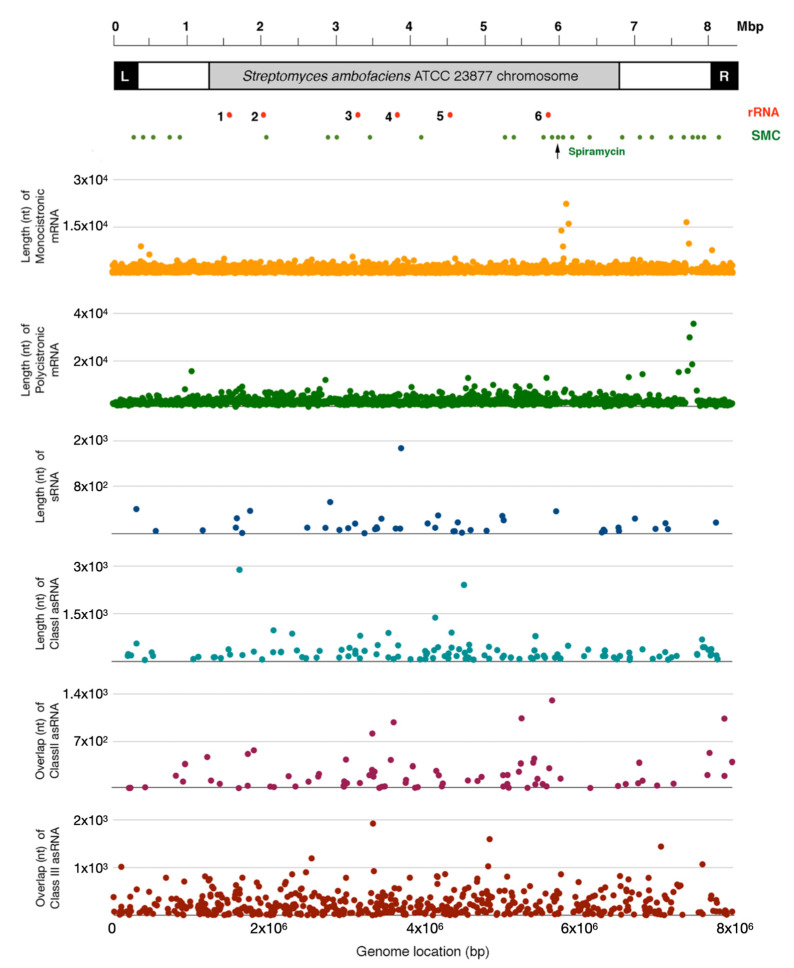
Genomic map showing the distribution of monocistronic and polycistronic transcription units, and ncRNAs’ loci in the linear chromosome of *S. ambofaciens* ATCC 23877.

Both polycistronic and monocistronic transcription units appeared to be uniformly distributed along the linear chromosome of *S. ambofaciens* as shown in Figure 1 that illustrates the transcription units as a function of their lengths and locations in the genome map. The median and length range of these transcription units are reported in Table 1. Some monocistronic and polycistronic transcription units largely exceeding the average length were located near the chromosome “right arm” (Figure 1). Other large monocistronic transcription units mapped to a specific “core” region of the *S. ambofaciens* chromosome where the spiramycin gene cluster and additional gene clusters for secondary metabolites are located. Computational analysis also reveals 162 new transcripts putatively originated from dedicated promoters. We classified them into sRNAs (Supplementary Table S3) and asRNAs (herein referred as asRNAs I) according to the fact that, based on our re-annotation, they overlap on the opposite strand of another gene (Figure 1, and Supplementary Table S4).

**Figure 2:**
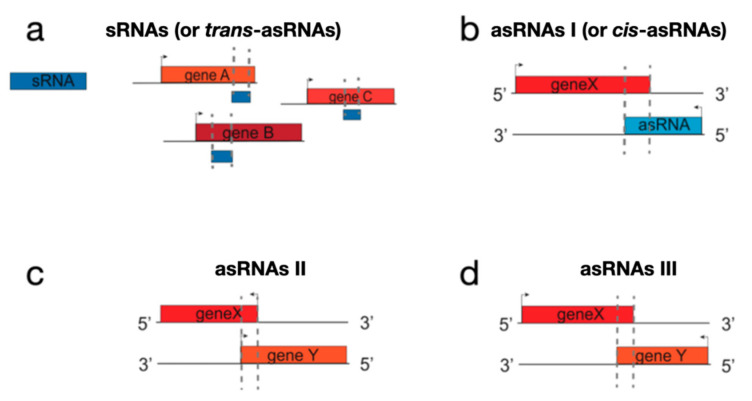
Scheme of ncRNA classes identified in this study. (**a**) sRNA; (**b**) asRNA I; (**c**) asRNA II; (**d**) asRNA III.

Furthermore, we identified asRNAs resulting from divergent transcription (herein referred to as asRNAs II) (Supplementary Table S5) and asRNAs resulting from the overlap of the terminal region of convergently oriented genes (cutoRNAs, herein referred to as asRNAs III) (Supplementary Table S6). A scheme of sRNA (Figure 2a), asRNA I (Figure 2b), asRNA II (Figure 2c), and asRNA III (Figure 2d) is depicted in Figure 2. Abundances and median plus length ranges of these three classes of asRNAs are reported in Table 1, while Figure 1 illustrates the asRNAs I, II, and III as a function of their lengths and locations in the genome map. For asRNA II and asRNA III, only the total overlaps between the two involved genes were considered. Most sRNAs and asRNAs I and II were mapped in the “core” region of the *S. ambofaciens* linear chromosome, at variance with asRNAs III that were uniformly distributed along the chromosome. The high number of asRNA III (also known as cutoRNAs) is noteworthy, a typical feature of streptomycetes that have genomes with high GC content and weak transcription termination [20]. We need to specify that our classification is based on only the transcriptional landscape and that open reading frames (ORFs) were not investigated among the newly identified transcripts; doing this a different classification could originate. The presence of some asRNAs I, II, and III with lengths largely exceeding the average lengths (Table 1) is also remarkable.

**Figure 3:**
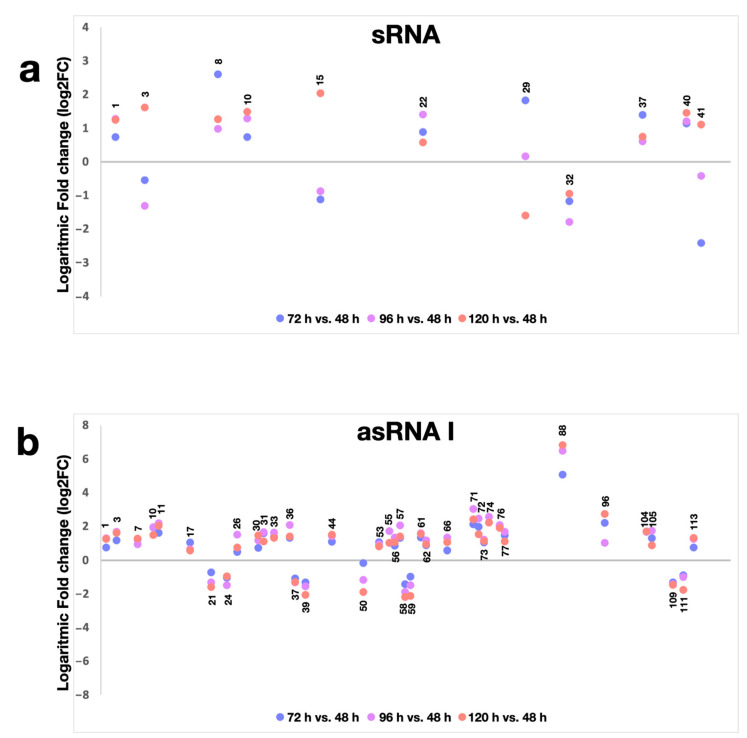
Differential expression of ncRNAs during the growth of *S. ambofaciens* ATCC 23877. (**a**,**b**) *S. ambofaciens* ATCC 23877 was cultivated in YS, and ncRNA levels were determined after 48 h, 72 h, 96 h, and 120 h (Supplementary Table S7). Log2Fold changes of sRNA (**a**) and asRNA I (**b**) resulting differentially expressed in at least one timepoint were reported. The sRNAs and asRNAs I are arranged on the x-axis according to the sRNA and asRNA I ID numbering reported in Supplementary Tables S3 and S4.

RNAseq data showed that 11 (out of 49, i.e., about 25%) sRNAs and 39 (out of 119, i.e., about 33%) asRNAs I exhibited clear differential expression during the growth of the *S. ambofaciens* ATCC 23877 strain (Figure 3; Supplementary Table S7). In particular, most of them (7 sRNAs and 31 asRNAs I) were upregulated in at least one of the later time points (72 h, 96 h, or 120 h) when compared to the 48-h expression, while showing an increasing trend for all the later time points in all cases. Conversely, only a few of them (4 sRNAs and 8 asRNAs I) were more expressed at 48 h than at later time points.

### 2.2. Novel sRNAs and Putative Targets

Only very few sRNAs with known functions have been functionally characterized in *Streptomyces*. In *Streptomyces coelicolor* A3(2), Vockenhuber et al. [23] identified 63 ncRNAs including 29 *cis-*encoded antisense RNAs and confirmed expression for 11 of them which are now included in Rfam database (scr1601, scr2736, scr2952, scr3202, scr3920, scr4115, scr4389, scr4632, scr5676, scr6106, and scr6925) and in prevalence show growth-phase dependent expression. Of these ncRNAs, 5 out of 11 (scr1601, scr2736, scr2952, scr3920, and scr4115) were evolutionarily conserved in more than 50% of tested *Streptomyces* [23].

The Rfam database (http://rfam.xfam.org/, accessed on 15 February 2020) was first used to search for functional information and the degree of phylogenetic conservation of predicted *S. ambofaciens* sRNAs (Supplementary Table S8). Computational analysis predicted scr2736 homolog (sRNA #10) among the sRNAs, in addition to other conserved sRNAs including the bacterial small signal recognition particle RNA (SRP RNA) (sRNA #20), and 6C RNA (sRNA #24). 6C RNA is a class of ncRNA present in actinomycetes, which is characterized by a conserved RNA structure having two stem-loop structures each containing six or more cytosine residues. Transcription of the *S. coelicolor* 6C RNA increases during sporulation [22]. In *Mycobacterium tuberculosis,* 6C sRNA regulates genes involved in various processes including DNA replication and protein secretion [41].

Extending our search to UTRs, we found scr1601, scr4115, scr4677, scr5239, and scr5676 homologs that were not predicted among sRNAs. This prediction also reveals conserved *cis*-acting regulatory elements including several riboswitches (S-adenosyl-methionine (SAM), glycine, flavin mononucleotide, thiamine pyrophosphate, cobalamin, cyclic di-AMP (*ydaO/yuaA*) riboswitches), *msiK* RNA motif [42], *che1* RNA motif [43], and the Group II catalytic intron D1-D4-7 (Supplementary Table S8). scr5239 is probably one of the currently best-characterized sRNAs in *Streptomyces coelicolor* [22,23,44]. scr5239 has two targets; it inhibits agarase DagA expression by direct base pairing to the *dagA* mRNA coding region [45], and it represses translation of methionine synthase *metE* mRNA by binding at the 5′ end of its open-reading frame [46].

sRNA scr4677 is located in the intergenic region between anti-sigma factor SCO4677 gene and a putative regulatory protein gene SCO4676. scr4677 expression requires SCO4677 activity and scr4677 itself seems to affect the levels of SCO4676 mRNA [47]. Both SCO4676 and SCO4677 affect the production of the blue-pigmented antibiotic actinorhodin under specific growth conditions [47]. In intracistronic sequences of operons Rfam search predicted scr3202, in addition to other conserved *cis*-acting regulatory elements including *raiA*-hairpin RNA motif [43], *nrdJ* RNA motif [43], Actino-*pnp* RNA motif [43], group II catalytic intron, and cyclic di-AMP (*ydaO/yuaA*) and cobalamin riboswitches (Supplementary Table S8).

Trying to understand the reason for the possible misclassification of scr1601, scr4115, scr4677, scr5239, scr5676, and scr3202 homologs in *S. ambofaciens* we went back to timepoint predictions in search of possible prediction errors (Supplementary Figure S1). Only for two of them (scr5239, scr5676), we found predictions for both UTR and ncRNAs in different days and relevant signals corresponding to the expected ncRNAs length, so we decided to add them to the list of sRNA as sRNA #44 and sRNA #45.

Starting from all 43 sRNA sequences (Supplementary Table S3) and from the 5 scrRNA sequences predicted in other ncRNAs (Supplementary Table S3), GLASSgo software [48,49,50] was used to analyze the degree of phylogenetic conservation of the predicted *S. ambofaciens* sRNAs. Results indicated that 26 out of 49 sRNAs (i.e., sRNA #5, sRNA #7–13, sRNA #15, sRNA #17, sRNA #19–20, sRNA #29, sRNA #31–35, sRNA #40–41, sRNA #44–49) are highly conserved in many *Streptomyces spp*. (Supplementary Table S9). To search for functional information about these predicted sRNAs, we used IntaRNA software [51,52]. IntaRNA allowed us to select a total of 241 putative target mRNAs for detected 49 sRNAs (Supplementary Table S10). The results indicated the potential of detected sRNAs to interact with more than one target mRNA. Conversely, there were also cases where a single mRNA is targeted by multiple sRNAs.

Most putative targets code for proteins involved in transcription control (including sigma and transcriptional factors). The case of Lrp (AsnC family) transcription factor mRNAs, which are targeted by multiple sRNAs, is noteworthy. IntaRNA predicted three sRNAs (sRNA #5, sRNA #15, sRNA #42) targeting Lrp paralog RS15615, one sRNA (sRNA #23) targeting Lrp paralog RS13995, and one sRNA (sRNA #41) targeting Lrp paralog RS05445 mRNAs. In addition to Lrp, AcrR (TetR family) transcription factor mRNAs were targeted by several sRNAs. In particular, AcrR (RS05365) mRNA was targeted by sRNA #3, AcrR (RS04560) mRNA by sRNA #8, AcrR (RS14270) mRNA by sRNA #11, and AcrR (RS15315) mRNA by sRNA #49.

Among housekeeping functions, the finding of 3 sRNAs may be noteworthy, each of which was predicted to target distinct DNA polymerase III subunits mRNAs (epsilon subunit RS07315, sRNA #2; alpha subunit RS08380, sRNA #3; delta’ subunit RS19530, sRNA #33). It may also be relevant to note the presence of one sRNA, sRNA #44, targeting both *infC* mRNA encoding translation initiation factor IF-1, and *rpsR* mRNA encoding 30S ribosomal protein S18, while two sRNAs, sRNA #33, sRNA #40, were predicted to target the *asnB* mRNA coding for asparagine synthetase B (RS16870).

A number of sRNAs were predicted to target the transcripts of structural genes involved in cell-wall assembly and morphological development, including two *ssgA* paralogs (RS07365; RS13975), coding for sporulation and cell division protein SsgA-like, whose transcripts are targeted by two sRNAs (sRNA #28, and sRNA #19, respectively), while the transcript encoding Cps2a (LytR-Cps2A-Psr (LCP) family), an anionic cell wall polymer biosynthesis enzyme, is targeted by sRNA #21 and sRNA #3). sRNA #4 is predicted to target *nanH* mRNA coding for sialidase. Several sRNAs were predicted to target the transcripts of genes involved in secondary metabolite production including sRNA #2 targeting *wecE* (RS26695) coding for dTDP-4-amino-4,6-dideoxygalactose transaminase in the spiramycin gene cluster; sRNA #10 targeting *fabH* (RS34875) coding for 3-oxoacyl-ACP synthase III in a type I PKS gene cluster; sRNA #13 targeting *lanB* (RS34475) coding for lanthionine biosynthesis dehydratase LanB; and sRNA #14 targeting *alpH* coding for O-methyltransferase paralogs RS00755 and RS35400 in duplicated alpomycin gene cluster.

Regarding the oxidative stress response, one may also note the presence of five sRNAs putatively targeting mRNAs of genes involved in iron and sulfur metabolism, which plays a role in redox balance, including sRNA #3 targeting *soxR* (RS35165) mRNA, sRNA #7 targeting *hemH* mRNA coding for protoheme ferro-lyase (ferrochelatase) (RS26415), sRNA #9 targeting *metC* mRNA coding for cystathionine beta-lyase/cystathionine gamma-synthase (RS22645), sRNA #36 targeting *mmuM* mRNA coding for homocysteine/selenocysteine methylase (RS27820), and sRNA #45 targeting mRNA of a gene (RS01550) coding for putative cysteine synthase (RS01550).

Overall, these results seem to associate most of *S. ambofaciens* sRNAs with gene functions involved in the control of mycelial growth, morphological differentiation, secondary metabolism, and stress responses. Indeed, one might note sRNA #2 targeting two paralogs coding for diadenosine tetraphosphate (Ap_4_A) hydrolase (RS00910 and RS35245). It may also be worth noting the presence of two sRNAs, sRNA #3 and sRNA #43, targeting a pSAM2-encoded protein (RS19305) harboring a GGDEF domain that is present in diguanylate cyclase (c-di-GMP synthetase) or its enzymatically inactive variants. In *Streptomyces* c-di-GMP is a central component of the signal transduction network by controlling the activity of the developmental master regulator BldD [53].

### 2.3. Antisense Transcription in S. ambofaciens

**Figure 4:**
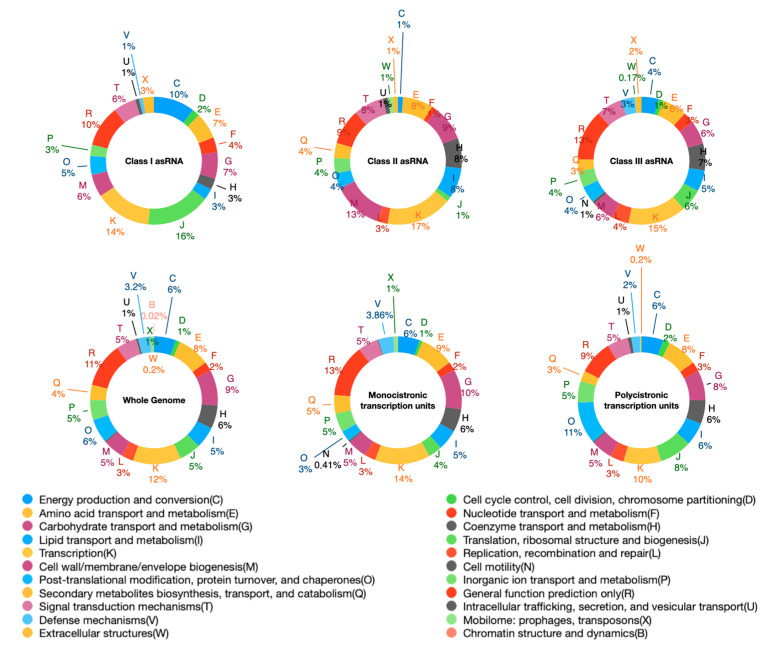
Functional classification of CDSs arranged in monocistronic or polycistronic transcripts and CDSs targeted by asRNAs (I, II, and III). CDSs that are predicted targets of asRNAs I, asRNAs II, and asRNAs III are classified according to Clusters of Orthologous Groups (COGs). As references, COGs classification of whole genome CDSs, and CDSs in monocistronic and polycistronic transcriptional units are also shown. Genes with unknown functions are excluded from the representation.

Although it was initially considered non-functional transcriptional noise, antisense transcription is increasingly considered important in regulating gene expression [10]. This assumption is corroborated by the high phylogenetic conservation of several asRNAs. In *S. ambofaciens* we detected 119 asRNAs putatively expressed from a dedicated promoter (asRNAs I) targeting 146 mRNAs (Supplementary Table S4). Two of them were antisense to tRNAs, seven to pseudogenes, three to other ncRNA, and two to mixed feature types. The remaining 133 targeted newly defined gene extensions (UTR and within_operon regions) (19), CDS (83), and a mix of the three (31). Only 21 antisense_ncRNA accounted for less than 90% of their length for the target TUs. Functional enrichment analysis by Clusters of Orthologous Groups (COGs) demonstrated that asRNA-targeted CDSs, with respects to whole-genome CDSs, showed a prevalence in the COGs J (translation, ribosomal structure, and biogenesis), F (nucleotide transport and metabolism), and C (energy production and conversion) (Figure 4).

GlassGO analysis revealed that about 30% of class I asRNAs were specific to *S. ambofaciens*: 10% of the sequences (12 out of 119) were identified only in *S. ambofaciens* ATCC 23877 strain while 20% of the sequences (24 out of 119) were identified in both ATCC 23877 and DSM 40697 strains. The remaining 70% of the sequences appeared to be evolutionarily conserved: 56.3% were present in the *Streptomyces* genus, and 13.4% in both *Streptomyces* and other genera (Supplementary Table S11). Although this tool does not allow assess whether an ncRNA is expressed or not, the results are accurate (see Section 4.7 of the Materials and Methods).

Among asRNA-targeted mRNAs of genes involved in energy production and conversion, we found *gltA2* (RS15735) coding for citrate synthase 2, *nuoF* (RS20780), *nuoL2* (RS21040), and *nuoM2* (RS21045). *nuoF*, *nuoL2*, and *nuoM2* code for subunits of the NADH:quinone oxidoreductase multi-protein complex (the respiratory complex I) (Supplementary Table S4). Like other streptomycetes, *S. ambofaciens* possesses a complete 14-subunit encoding *nuo* operon (*nuoA-N*), and an additional copy of many *nuo* genes (*nuoA2, nuoB2, nuoD2, nuoH2, nuoI2, nuoK2, nuoL2, nuoM2,* and *nuoN2*). While *nuoD2* is separated from the other *nuo* genes, the other ones, i.e., *nuoA2, nuoB2, nuoH2, nuoI2, (YnuoJ) nuoK2, nuoL2, nuoM2,* and *nuoN2* are clustered together in an operon (*nuo2*). Interestingly, the asRNAs targeting *nuoF*, *nuoL2*, and *nuoM2* are evolutionarily conserved in streptomycetes [20]. Two asRNAs that appear to be conserved in *Streptomyces*, albeit not described so far in this genus, map in the biosynthetic gene cluster for aminobacteriohopanetriol (a C35 hopanoid), while another asRNA is transcribed complementary to *dnaK* mRNA.

We found a remarkable number of asRNAs having as putative targets, mRNAs of genes involved in translation, ribosomal structure, and biogenesis. Overall, these genes account for 16% of all targets, including: i.) ten 30S ribosomal protein-encoding genes *rpsA* (S1) (RS09750), *rpsF* (S6) (RS17780), *rpsH* (S8) (RS21400), *rpsI* (S9) (RS21495), *rpsL* (S12) (RS21225), *rpsM* (S13) (RS21455), *rpsM2* (S13) RS07055, *rpsO* (S15) (RS25835), *rpsP* (S16) (RS25395), and *rpsT* (S20) (RS12280); ii.) six 50S ribosomal protein-encoding genes *rplA* (L1) (RS21180); *rplK* (L11) (RS21175); *rplM* (L13) (RS21490); *rplU* (L21) (RS12440); *rpmG* (L33) (RS21110); and *rpmJ* (L36) (RS21450); iii.) *infA* encoding translation initiation factor IF-1 (RS21445); and iv.) phenylalanine-tRNA ligase subunit beta and alpha-encoding genes, respectively, *pheT* (RS07660) and *pheS* (RS07665). Among asRNAs having as putative targets, mRNAs of genes encoding proteins of the transcription machinery, the presence of two asRNAs may be relevant, that are transcribed complementary, respectively, to *nusA*, coding for the transcription elongation factor NusA, and *cdnL*, coding for an AmiR and NasR Transcriptional Antiterminator Regulator domain (ANTAR)-containing protein.

In addition to r-protein genes, we detected several asRNAs that are transcribed complementary to the 5 tRNAs, i.e., tRNA^Cys^ (GCA, codon UGC) (RS28910), tRNA^Leu^ (CAG, codon CUG) (RS28915), tRNA^Pro^ (UGG, codon CCA) (RS28920), tRNA^Gly^ (CCC, codon GGG) (RS18700), and tRNA^Met^ (CAU, codon AUG) (RS21105) (Supplementary Table S4). One of the 5 tRNAs recognizes a specific codon for proline (CCA) that is less represented in *Streptomyces* CDSs with respect to other codons specifying the same amino acids (Supplementary Figure S2).

We found many putative asRNAs that are transcribed complementary to genes coding for regulatory proteins that oversee morphological and biochemical differentiation. In particular, an asRNA is transcribed complementary to *phoR* (RS16370) coding for the sensor kinase of the two-component PhoR-PhoP system [54]. Moreover, another asRNA is transcribed complementary to *whiB* coding for the regulatory protein WhiB that is essential for morphological differentiation in *Streptomyces* [55,56]. In addition, some asRNAs appear to also target genes coding for proteins involved in the control of secondary metabolite biosynthesis, and, in particular, spiramycin production and resistance. Precisely, asRNAs #88 is transcribed complementary to *smrR* mRNA (Supplementary Table S4). As mentioned above, *smrR* codes for a transcriptional activator of the spiramycin gene cluster [57,58]. Another asRNA, asRNAs #87, is transcribed complementary to *smrB* mRNA (Supplementary Table S4).

### 2.4. Expression of ncRNAs in S. ambofaciens ATCC 23877, and Derivative ΩpirA and ΩpirA rpoB(R) Mutants

With the aim of gaining some insight into the possible biological effects of predicted ncRNAs, we analyzed their expression levels in three *S. ambofaciens* strains: the wild type ATCC 23877 strain (hereafter indicated as w.t.), an isogenic *pirA*-defective mutant (here referred to as ΩpirA) that is characterized by central carbon and energy metabolism imbalance, high sensitivity to oxidative injury, and repression of polyketide antibiotic production [35], and a *pirA*-defective derivative strain harboring a stringent RNA polymerase exhibiting partial suppression of *pirA*-defective phenotypes (here referred to as ΩpirA rpoB(R)). Specifically, in the ΩpirA strain the integrative plasmid pTYM-18 (a ΦC31 Att/Int system-based vector) inactivates *pirA*, encoding a redox-sensitive negative modulator of very long-chain acyl-CoA dehydrogenase, which catalyzes the first committed step of the beta-oxidation pathway [35]. 

In ΩpirA rpoB(R) the integrative plasmid pTYM-18 was replaced by pTYM-rpoB(R), a pTYM-18 derivative harboring the *rpoB*(R) gene of *Nonomuraea gerenzanensis* ATCC 39727. *N. gerenzanensis* possess two RNA polymerase beta-chain genes, *rpoB(S)* (the wild type *rpoB* gene), and *rpoB(R)* (the mutant type *rpoB* gene) [59].

**Figure 5:**
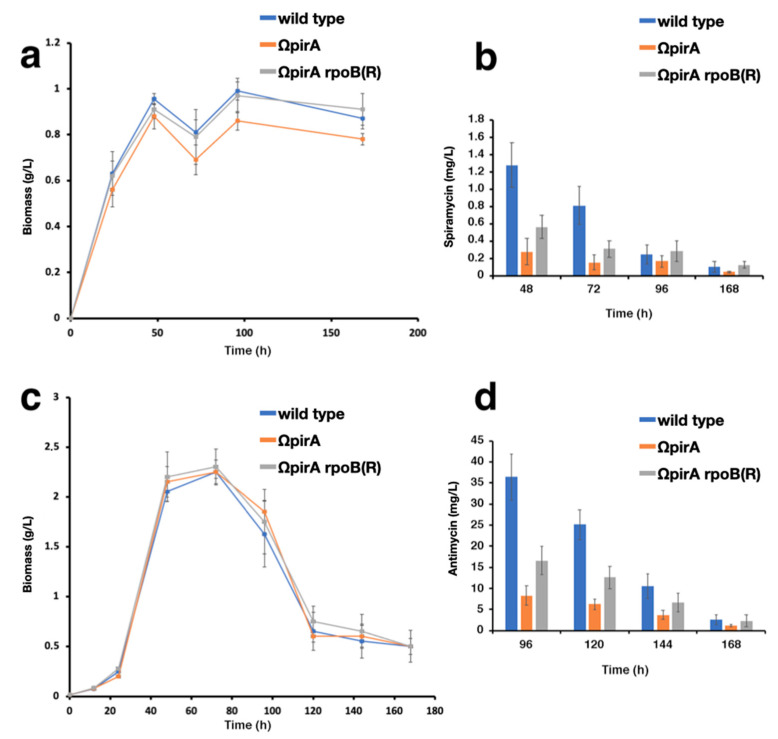
Growth curves and antibiotic production in *S. ambofaciens* ATCC 23877 (w.t.), and derivative ΩpirA, and ΩpirA rpoB(R) mutants. (**a**–**d**) Strains were cultivated in either YS (**a**,**b**) or SFM (**c**,**d**) broths, and biomass (**a**,**c**), spiramycin (**b**), and antimycin (**d**) values were determined at different time points. The values were calculated as the mean of three or more experiments. The errors bar indicates the standard deviation.

RpoB(R) harbors a specific histidine-to-asparagine substitution in the rifampin resistance cluster I, which was believed to be essential for the activation of the secondary metabolism by mimicking a “stringent” phenotype [36,37], and, indeed, we found that *rpoB(R)* in *S. ambofaciens* ΩpirA increased spiramycin (in YS medium) (Figure 5a,b) and antimycin (in SFM medium) (Figure 5c,d) production. This effect may be a consequence of *rpoB(R)*-mediated phenotypic suppression of the “relaxed” phenotype exhibited by ΩpirA strain that was characterized by upregulation of genes involved in translation, ribosomal structure and biogenesis, and amino acid biosynthesis, and downregulations of *spoT*, encoding the enzyme involved in (p)ppGpp biosynthesis [35].

**Figure 6:**
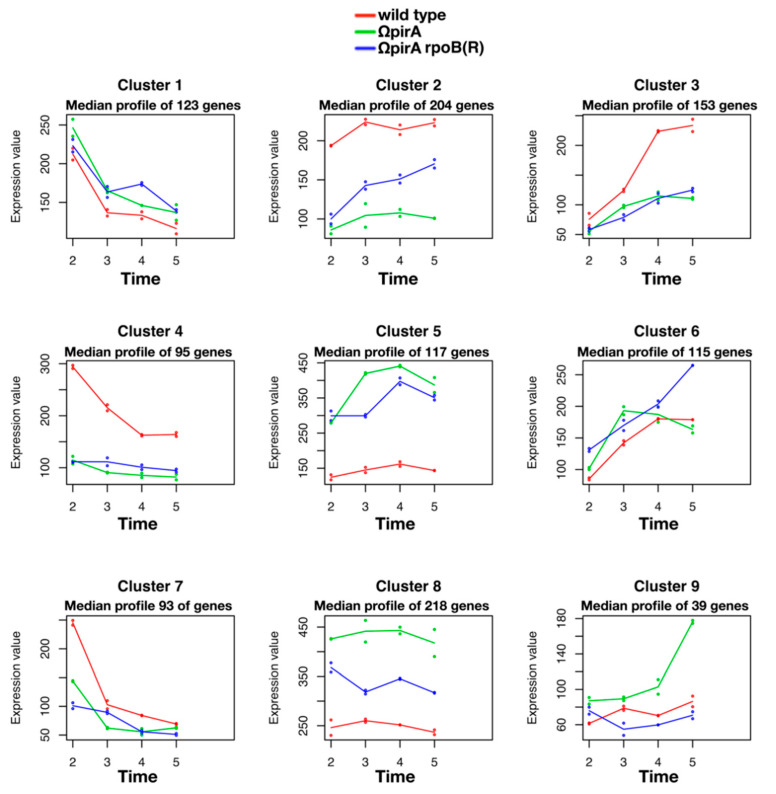
Time-course cluster analysis of RNAseq data in *S. ambofaciens* ATCC 23877 (w.t., red), and derivative ΩpirA (green), and ΩpirA rpoB(R) (blue) mutants. maSigPro identified the expression pattern of 1166 variable genes during the time course subdivided into 9 clusters (Supplementary Table S12) and median profile of each cluster was inferred from the expression patterns.

Partial suppression of *pirA*-associated phenotypes was also evident at the transcriptome level (Supplementary Table S12). In these experiments, w.t., ΩpirA, and ΩpirA rpoB(R) were cultivated in YS broth. In this medium, growth rates during rapid growth phase 1 (RG1), and final biomass values of ΩpirA were slightly lower as compared to w.t., while in ΩpirA rpoB(R) and w.t. these parameters were similar (Figure 5a). This finding was also supported by maSigPro analysis of RNA-Seq time series dataset that identified the expression pattern of 1166 of the most variable genes during the time course subdivided into 9 clusters (Supplementary Table S12). The median profile of each cluster was inferred from the expression patterns and was used to represent the expression profile of the strains (Figure 6). For most of the clusters, the expression profile of ΩpirA rpoB(R) shows an intermediate profile between w.t. and ΩpirA if not a complete rescue of either the expression levels, time course profile, or both, as in cluster 8 and in cluster 4, supporting the experimental data.

**Figure 7:**
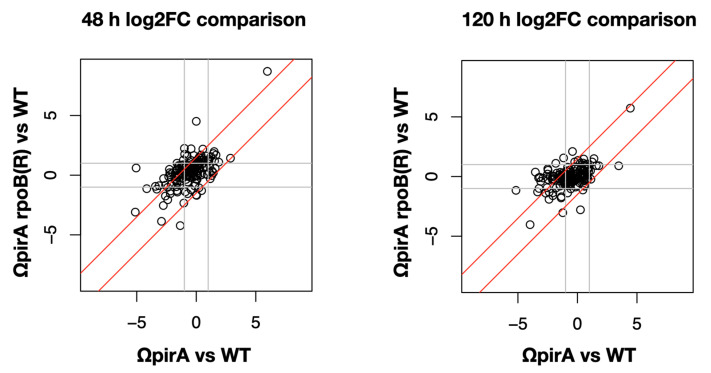
Differential expression of sRNas and asRNAs of class I in *S. ambofaciens* ATCC 23877 (w.t.), and derivative ΩpirA and ΩpirA rpoB(R) mutants at 48 h (left panel) and 120 h (right panel). (Supplementary Table S13). Log2fold changes of the two mutants compared to the w.t. strain were reported in the plots as indicated by the labels.

The growth of *Streptomyces* follows a biphasic curve in which two phases of rapid growth (RG1, RG2), interspersed by a transition phase (T), precede the stationary phase (S). ncRNA levels were compared in w.t., ΩpirA, and ΩpirA rpoB(R) at two corresponding time points: 48 h, RG1 and 120 h, stationary phase. Results demonstrated that 21 (out of 45, i.e., about 47%) sRNAs and 72 (out of 119, i.e., about 70%) asRNAs (asRNAs I) were differentially expressed at least in one strain when compared to the w.t. (Supplementary Table S13). The plots in Figure 7 show log2fold changes (log2FC) of the mutants compared to w.t. at 48 and 120 h. The data indicated that at 48 h more ncRNAs were differentially expressed in ΩpirA rpoB(R) with respect to 120 h (42 and 16, respectively), while for ΩpirA the number was similar (55 and 52, respectively). At 48 h the cloud was shifted up toward ΩpirA rpoB(R) indicating that, in general, the delta in expression level was higher for this strain, while at 120 h this was not true anymore. At both time points we could notice that the greatest part of differentially expressed genes was not proportional between the two mutants (not within the red lines) but tended to be differentially expressed only in ΩpirA genotype. sRNAs are generally equally distributed between differentially up and downregulated features, apart from ΩpirA rpoB(R) at 120 h which shows only 3 upregulated sRNAs (Supplementary Table S13). asRNAs showed clear trends of downregulation in mutant strains (85% to 97% of the differentially expressed features, Supplementary Table S13). Furthermore, ΩpirA rpoB(R) had an opposite trend at 48 h with 62% of upregulated sRNAs.

In the ΩpirA strain, a marked downregulation of all asRNAs I targeting genes involved in translation, ribosomal structure and biogenesis (i.e, asRNA #14, asRNA #20, asRNA #27, asRNA #28, asRNA #65, asRNA #66, asRNA #67, asRNA #68, asRNA #82, and asRNA #84) may be noted, while in ΩpirA rpoB(R) expression of these asRNAs was restored to w.t. levels (Supplementary Table S13). asRNAs that are transcribed complementary to tRNA^Met^ (CAU) (asRNA #63) were also downregulated in the ΩpirA strain, and their expression was restored to w.t. levels in ΩpirA rpoB(R). In contrast, asRNA #94, which is transcribed complementary to tRNA^Cys^ (GCA), tRNA^Leu^ (CAG), and tRNA^Pro^ (UGG), was more expressed in ΩpirA with respect to the w.t. strain.

As for asRNAs I targeting genes involved in secondary metabolism, we found that asRNA #119 was less expressed in ΩpirA than in both w.t. and ΩpirA rpoB(R). This asRNA is transcribed complementary to *afsA* coding for A-factor biosynthesis protein AfsA. Expression of asRNA #76, which was transcribed complementary to *whiB*, exhibited a similar trend. This asRNA was more expressed in w.t. than in ΩpirA, and even more in ΩpirA rpoB(R). In addition, asRNA #87, which is transcribed complementary to *smrB* in the spiramycin gene cluster, was also more expressed in w.t. than in ΩpirA, even more in ΩpirA rpoB(R) at earlier time point. *smrB* encodes for ribosomal protection factor that is involved in spiramycin resistance [60,61]. Therefore, it would be interesting to understand if there is a link between spiramycin production and expression of asRNA #87.

## 3. Discussion and Conclusions

In this study, the redefinition of monocistronic and polycistronic transcription units, and other structural elements including 5′- and 3′-UTR and intercistronic regions of the *S. ambofaciens* ATCC 23877 transcriptome was preliminary to the identification of novel sRNAs and asRNAs. We identified 45 sRNAs and 119 asRNAs (asRNAs I) that are predicted to be transcribed from dedicated promoters. Some sRNAs were previously identified in *Streptomyces*. Other sRNAs are novel, and bioinformatic analysis predicted several mRNAs encoding proteins involved in the control of mycelial growth, morphological differentiation, secondary metabolism, and stress responses, which may be targeted by these sRNAs. Indeed, most putative sRNA targets code for key proteins involved in transcription control including sigma and transcriptional factors.

Among the sRNAs that were predicted to target genes encoding transcriptional factors, it may be worth noting the cases of the sRNAs targeting the pleiotropic Lrp and AcrR paralogs. Specifically, the Lrp RS15615 was predicted to be targeted by 3 sRNAs. Lrp RS15615 is the homolog of SCO3361 in *S. coelicolor* A3(2). SCO3361 functions as a pleiotropic regulator controlling secondary metabolism and morphological development [62]. In particular, it activates actinorhodin (Act) production by directly binding to actII-ORF4 promoter, and it stimulates the expression of *amfC*, *whiB*, and *ssgB* thus promoting hyphae formation and sporulation [62]. Phenylalanine and cysteine were identified as the effector molecules of SCO3361, with phenylalanine reducing the binding affinity, whereas cysteine increasing it [62].

In this regard, one might note that 3 Hfq-dependent sRNAs, namely, DsrA, MicF, and GcvB, each independently downregulate the *lrp* transcript in *E. coli* [63]. MicF and DsrA interact with an overlapping site early in the *lrp* ORF, while GcvB acts upstream in the long lrp 5′-UTR [63]. In particular, GcvB was responsible for *lrp* downregulation in response to oxidative stress. Four AcrR paralogs appeared to be, instead, each targeted by a distinct sRNA. AcrR is a one-component allosteric repressor of the genes associated with lipid transport and antibiotic resistance. AcrR contains a C-terminal ligand-binding domain and an N-terminal operator-binding region. When fatty acid ligands bind to the C-terminal domain, a conformational change in the N-terminal domain is triggered, which releases the repressed DNA and initiates transcription [64].

Among the sRNAs that were predicted to target genes involved in the biosynthesis of cellular alarmones, one might note sRNA #2 targeting two paralogs coding for diadenosine tetraphosphate (Ap_4_A) hydrolase (RS00910 and RS35245). Ap_4_A is a product of the back reaction of the amino acid activation catalyzed by some aminoacyl-tRNA [65]. While on one hand, Ap_4_A may be considered an unavoidable and toxic by-product of protein synthesis that has to be cleared from the cell, on the other hand, it can function as a signal molecule and be deeply involved in the regulation of DNA replication, cell division, and stress response [66]. Ap_4_A hydrolases catalyzes the hydrolysis of Ap_4_A into two ADP and are involved in heat shock and oxidative stress responses in bacteria by regulating the intracellular Ap_4_A concentration [67].

Regarding the asRNAs that we detected in our study, some of them are also well conserved in *Streptomyces*, including the subgroup transcribed complementary to mRNAs coding for several subunits of the respiratory complex I. In particular, the asRNAs targeting *nuoF*, *nuoL2*, and *nuoM2* are evolutionarily conserved in streptomycetes [20]. The asRNA targeting *nuoF* (and the contiguous *nuoE* in some streptomycetes) was hypothesized to play a role as a “checkpoint” during complex I assembly [20], while the asRNAs targeting *nuoL2* and *nuoM2* were proposed to regulate the differential assembly of NuoL vs. NuoL2 and NuoM vs. NuoM2 into the respiratory complex I [20], similarly to what was observed in several cyanobacteria in which the multiplicity of functions assigned to cyanobacterial complex I (respiration, cyclic electron flow, and CO_2_ uptake) relies upon the diversity of the NdhD (NuoM) and NdhF (NuoL) protein isoforms resulting in the occurrence of distinct complex I assemblages [68].

Noteworthy, we found a remarkable number of asRNAs having as putative targets mRNAs of genes involved in translation, ribosomal structure, and biogenesis, in addition, two asRNAs that are transcribed complementary, respectively, to *nusA*, coding for the transcription elongation factor NusA, and *cdnL*, coding for an ANTAR-containing protein. To our knowledge, asRNAs targeting ribosomal protein (r-protein)-encoding genes have not been described so far in *Streptomyces*. However, a survey of small ncRNAs associated with r-protein operons in the bacterial pathogens *Staphylococcus aureus*, *Vibrio cholerae*, *Salmonella enterica* sv. Typhi, and *Mycobacterium tuberculosis* reported the presence of a total of 13 asRNAs transcribed complementary to nine r-protein mRNAs [69].

The physiological significance and the molecular mechanism by which these asRNAs can modulate the expression of the r-protein-encoding genes are currently under investigation. In all living organisms, ribosome assembly is subject to extensive feedback regulation to ensure correct ribosome manufacture in response to a variety of environmental and metabolic changes. In bacteria, despite numerous investigations, the mechanisms for the ribosome feedback regulation, and growth rate-dependent regulation of rRNA synthesis in bacteria other than the model organisms *Escherichia coli* remain to be elucidated.

Studies in *E. coli* demonstrated that r-protein synthesis is tightly regulated by numerous mechanisms including translational coupling, translation repression, or premature transcription termination [70]. In particular, in *E. coli* expression of more than half of the r-protein genes are controlled by 12 distinct RNA autogenous regulatory elements by which these proteins inhibit the translation of their own mRNA [71]. However, only two or three of these regulatory elements are widely distributed across many bacteria phyla raising some concerns about the full transferability of the r-protein regulation model established in *E. coli* to other bacteria, including the streptomycetes [71,72,73]. The discovery of asRNAs targeting so many r-protein-encoding genes in *S. ambofaciens* may suggest the existence of an additional regulatory level.

In addition to r-protein genes, we detected several asRNAs that are transcribed complementary to the 5 tRNAs were also detected. To our knowledge, this finding is unprecedented in *Streptomyces*. Moreover, it may be interesting to note that one of the 5 tRNAs recognize a specific codon for proline (CCA) that is less represented in *Streptomyces* CDSs with respect to other codons specifying the same amino acids (Supplementary Figure S2).

*Streptomyces* use rare codons to regulate their physiology. The most known example is *bldA* that codes for tRNA^Leu^ (UAA) that recognizes a rare leucine codon, UUA (about 0.01% of codons). The tRNA^Leu^ (UAA) is unnecessary for vegetative growth but is required for some aspects of secondary metabolism and morphological development [74]. In streptomycetes, about 3% of genes contain a UUA codon, which is mostly associated with either recently acquired genes by horizontal gene transfer, or genes coding for pleiotropic or pathway-specific regulators of secondary metabolism and morphological development [74]. Expression levels of these genes are strictly dependent on tRNA^Leu^ (UAA) levels, which increase during the stringent response [74]. In *S. ambofaciens*, UUA codons are present in *srmR* (RS26685) (containing 1 UUA codon) and *srmS* (RS26595) (containing 3 UUA codons) genes encoding two pathway-specific regulators of the spiramycin biosynthetic cluster. It is presumable that tRNA^Pro^ (UGG) may have a similar regulatory function on secondary metabolism and morphological differentiation. This hypothesis is supported by a considerable higher frequency of this codon in some CDSs for proteins involved in secondary metabolism and morphological development control, including *bldG* (RS24355) (Supplementary Figure S2). The existence of an asRNA that is transcribed complementary to the tRNA^Pro^ (UGG) gene could add complexity to this putative regulatory level.

The association between antisense transcription and *Streptomyces* developmental cycle seems to be supported by the evidence of many putative asRNAs that are transcribed complementary to genes coding for regulatory proteins that oversee morphological and biochemical differentiation. In particular, two asRNAs are transcribed complementary to *phoR* and *whiB*, respectively. PhoR is the sensor kinase of the two-component PhoR-PhoP system that controls both primary and secondary metabolism in *Streptomyces* in response to phosphate availability [52], while the regulatory protein WhiB, which belongs to the Wbl family of proteins that are characterized by an (4Fe-4S) iron-sulfur cluster, is essential for sporulation septation. By acting in concert with WhiA, WhiB halts the aerial growth to initiate the septation event at the aerial mycelial tip, and chromosome partition into the spores [53,54]. In *S. coelicolor* A3(2) WhiA and WhiB function cooperatively to control the expression of a common set of genes organized in about 240 transcriptional units [54]. Both *whiA* and *whiB* transcription is subject to negative repression by BldD when this regulatory protein is associated with c-di-GMP, whose levels are sustained high during vegetative growth [75].

Some asRNAs appear to also target genes coding for proteins involved in the control of secondary metabolite biosynthesis, and, in particular, spiramycin production and resistance. An asRNA is transcribed complementary to *smrR* mRNA. SmrR is a key transcriptional activator of the spiramycin gene cluster [55,56]. SrmR acts by activating, in turn, the pathway-specific transcriptional activator SrmS that controls most of the spiramycin biosynthetic genes [56]. SrmR is characterized by Hsp70 superfamily and GAF superfamily domain at the N-terminus, and PucR-like helix-turn-helix domain (HTH_30) at the C-terminus. The GAF domain is a type of protein domain that was so named after its initial discovery in cGMP-specific phosphodiesterases, adenylyl cyclases, and FhlA (formate hydrogen lyase transcriptional activator). This universal domain is responsible for binding allosteric regulatory molecules such as the second messenger cyclic nucleotides cGMP and cAMP [76], although in some bacterial proteins the GAF domain was shown to contain haem [77] or a non-haem mononuclear iron center [78] enabling them to sense molecular oxygen or nitric oxide, a second messenger gaseous compound whose importance in the physiological transitions that led to morphological differentiation in *Streptomyces* is increasingly apparent [79]. Another asRNA is transcribed complementary to *smrB* mRNA *smrB* gene (RS26680) is adjacent to *srmR* (RS26685) and transcribed in convergent direction and encodes an ABC-F type ribosomal protection protein [61], SrmB, that is involved in spiramycin resistance [60]. Further work is required to understand if and to which extent these asRNAs, targeting *srmR* and *srmB,* may contribute to either regulation of spiramycin production, resistance, or both.

The availability of a catalog of sRNAs and asRNAs in *S. ambofaciens* led us the opportunity of analyzing their expression in particular genetic backgrounds to gain some insights on the interplay between non-coding RNA transcription, stringent/relaxed phenotype, and antibiotic production. It was previously suggested that the repressed polyketide antibiotic biosynthesis in ΩpirA may be the consequence of a direct effect of the metabolic imbalance due to the lack of PirA, a redox-sensitive modulator of beta-oxidation, leading to a modified carbon flux between glycolysis, Krebs cycle, ethylmalonyl-CoA pathway, and lipid metabolism, with an increased and deregulated flow through the beta-oxidation and compensatory lipid biosynthesis and accumulation of lipid esters [35]. This modified carbon flux may be responsible for changes in the intracellular concentration of short-chain acyl-CoA pools that are precursor monomers for polyketide antibiotic biosynthesis. Here, we provide evidence that the repressed antibiotic biosynthesis in ΩpirA may be also associated with its “relaxed” phenotype that may be an indirect consequence of the modified carbon flux. Indeed, the stringent response, which negatively controls the expression of genes involved in translation, ribosomal structure and biogenesis, amino acid and nucleotide biosynthesis, is also essential to activate morphological and biochemical differentiation in streptomycetes [80,81]. The “relaxed” phenotype of ΩpirA is suppressed in ΩpirA rpoB(R) expressing a “stringent” RNA polymerase [58,59].

**Figure 8:**
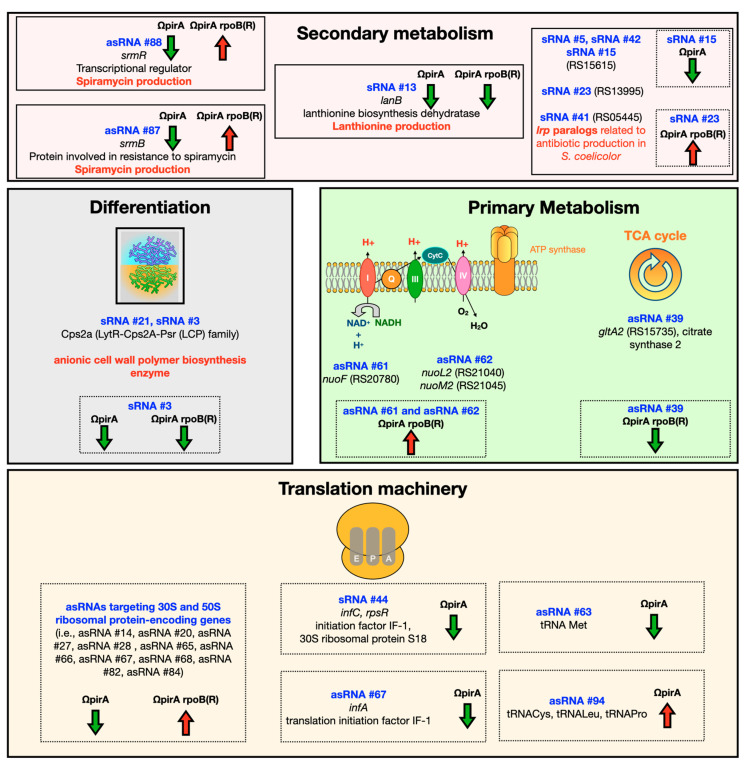
A summarized scheme depicting the up or downregulated ncRNA involved in secondary metabolism, morphological development, primary metabolism, translation, and ribosomal structure.

Intriguingly, we found a considerable number of asRNAs I having as targets mRNAs involved in translation, ribosomal structure, and biogenesis and showed that most of these asRNAs are differently modulated in w.t., ΩpirA, and ΩpirA rpoB(R), with downregulation in ΩpirA, and upregulation in w.t., and even more in ΩpirA rpoB(R). A summarized scheme depicting the up or downregulated ncRNA involved in above mentioned processes has been reported in Figure 8. These findings open the possibility that the expression of these asRNAs may be stringently controlled, and that, in turn, these asRNAs may be involved in the negative control of genes involved in translation, ribosomal structure, and biogenesis, consistent with RNAseq data, thereby overseeing critical aspects of microbial physiology.

## 4. Material and Methods

### 4.1. Bacterial Strains, Media and Growth Conditions

*S. ambofaciens* ATCC 23877 was obtained from the American Type Culture Collection (ATCC). The genome sequence of this microorganism was determined [33]. Derivative strain *pirA*::pTYM-18 (here indicated as ΩpirA) was previously described [35]. This strain was obtained by introducing the shuttle-plasmid pTYM-18, which integrates into *pirA* gene, into *S. ambofaciens* ATCC 23877 by conjugation *with E. coli* GM2929/pUB307::Tn7, as described previously [34]. Derivative strain *pirA*::pTYM-rpoB(R) (here indicated as ΩpirA rpoB(R)) was obtained by introducing the shuttle-plasmid *pirA*::pTYM-rpoB(R) already described [59] into *S. ambofaciens* ATCC 23877 by conjugation as described above.

The composition (per liter) of the media used in this study for *S. ambofaciens* growth and manipulation is here reported. Yeast starch (YS) broth: 2 g yeast extract, 10 g soluble starch, (18 g agar in YS agar); SM-II: 15 g dextrose, 10 g soybean flour, 0.5 g MgSO_4_·7H_2_O, 5 g CaCO_3_, 15 g agar; soya flour mannitol (SFM) agar: 20 g mannitol, 20 g soya flour, 20 g agar. The composition of R2 agar is reported [82]. For fermentation experiments, *S. ambofaciens* strains were cultivated in shake flasks at 28 °C on a rotary shaker at 200 rpm as described [34,83].

### 4.2. Spiramycin Production Assay

Spiramycin production by *S. ambofaciens* broth cultures was assessed by high-performance liquid chromatography-electrospray ionization-mass spectrometry (HPLC-ESI-MS) as described [83]. At different time intervals, supernatants were filtered through Phenex-RC membrane (0.45 mm; Phenomenex). Five μL of a solution of erythromycin (1 mg/mL) (Sigma-Aldrich) in 30% v/v aqueous acetonitrile plus 210 μL of acetonitrile were added to 500 μL of filtrated samples; mixture samples were vortexed, centrifuged at 4 °C for 5 min, and then 2 μL of the supernatant were injected for spiramycin determination.

The high-performance liquid chromatography (HPLC)-ESI-MS apparatus consisted of a Surveyor MS quaternary pump coupled to a Finnigan LCQ Deca XP Plus mass spectrometer (ThermoFisher, Monza, Italy) equipped with an ESI source and a quadrupole ion trap analyzer. Xcalibur software (ThermoFisher) was used for instrument control and data analysis. The spectrometer was calibrated externally with a mixture of caffeine, MRFA and Ultramark (ThermoFisher) [84]. The mass spectrometer operated in the positive-ion mode with the following settings: sheath gas, 60 units; auxiliary gas, 20 units; spray voltage, 1.5 kV; capillary temperature, 325 °C; and capillary voltage, 10 V. Mass spectra were recorded in full-scan MS in the *m/z* range 400–1100. Spiramycin I, II and III were resolved on a BioBasic C-18 analytical column (150 × 2.1 mm, particle size: 5 μm) (Thermo Scientific, Monza, Italy), which was eluted with 5 mM ammonium formate, pH 7.0 (solvent A) and LCMS-grade acetonitrile (solvent B), at a flow rate of 200 μL/min, applying the following gradient of solvent B: 0 min, 15%; 1 min, 15%; 15 min, 70%; 20 min, 70%; 21 min, 15%; and 25 min, 15%. To minimize source fouling during analysis, the eluent was directed to the source only during erythromycin and spiramycin elution by using the divert valve on the mass spectrometer. Calibration curves were made based on peak area ratios of analyte/internal standards vs. analyte concentrations, using 6 point calibration standards in the range of 0.1–5.0 μg/mL (0.1, 0.2, 0.5, 1.0, 2.5, and 5.0 μg/mL). The recoveries of the method, evaluated by spiking the samples with low (200 ng) and high (2 μg) levels of the analyte, were 97 and 93%, respectively.

The method limits of detection (LODs) and limits of quantification (LOQs) were determined using the samples fortified at the lower validation level. LODs and LOQs determined at S/N ratios of 3 and 10 for spiramycin were 20 and 67 ng/mL, respectively. 

### 4.3. Antimycin Production Assay

Bacteria were cultivated in SFM broth. At different time points, 1 mL of cultivation broth was collected, lyophilized, and then dissolved in 1 mL methanol. After centrifugation at 10,000× *g*, the pellet was discarded, and the supernatant was collected for analysis. Antimycin was analyzed by HPLC using a System Gold programmable solvent module 125 (Beckman) equipped with a Nucleosil C8 analytical column (200 × 4.6 mm; particle size: 3 µm, pore dimension: 120 Å) (Macherey & Nagel) maintained at 25 °C, and a UV detector (350 nm). The column was eluted with water (solvent A) and methanol (solvent B), at a flow rate of 1 mL/min, applying the following gradient of solvent B: 0 min, 10%; 20 min, 100%; 34 min, 100%; and 44 min, 10%. Pure antimycin (Sigma Aldrich) was used as standard.

### 4.4. RNA Extraction and RNAseq Experiments

*S. ambofaciens* strains w.t., ΩpirA, and ΩpirA rpoB(R) were grown in YS medium at 28 °C on a rotary shaker at 200 rpm. Four different time points (48 h, 72 h, 96 h, and 120 h) were collected for each strain in biological duplicates. Total bacterial RNA was extracted from the pellets, ribosomal RNAs were depleted, and sequencing libraries prepared as already described [34]. Each library was then sequenced on a MiSeq Illumina sequencer and 76 bp paired-end reads were produced.

### 4.5. S. ambofaciens Transcriptome Re-definition, ncRNA Prediction and Antisense Transcripts Definition on the Main Chromosome

We adopted Rockhopper [38,39,40] to obtain w.t. *S. ambofaciens* transcriptome redefinition and ncRNAs prediction using paired-end strand-specific RNA-seq already published [34]. The predictions were carried out at 48 h, 72 h, 96 h, and 120 h timepoints separately and, for each of them, we obtained the annotated genes list (eventually extended for the length of their UTRs) and ncRNAs one. We then pulled together daily predictions and we merged features resulting to overlap on the same strand both for ncRNAs and annotated genes lists, to reduce small inconsistencies in start and end position definition. Finally, we pooled together the two lists giving priority to UTRs prediction in case of inconsistencies, and we evaluated the distances from the nearest element on the same strand for each feature and eventual antisense overlaps using BEDTools functions [85].

This led us to highlight some peculiarities of Rockhopper [38,39,40] predictions that could be improved:

(1) The algorithm tends to split long UTRs into one UTR and one or more “predicted ncRNA”, usually separated by few or even no nucleotide between them. The same happens to long ncRNAs which are split into multiple close records. Tracks inspection instead shows no difference in coverage between the two features and no gaps.

(2) There were hotspots of ncRNAs predictions in rRNAs regions and on the arms of the main chromosome. These regions result from genome inverted repetitions which are particularly uncertain for predictions as reads are forced to map to one of the possible locations resulting in false antisense transcription predictions.

For this reason, we decided to merge all the predicted features found on the same strand at 15 or fewer nucleotides from each other, to manually collapse ncRNAs located within 500 nucleotides from one other when RNA-coverage tracks show continuous signal, and to exclude ncRNAs predicted from duplicated genes/regions to produce a sound *S. ambofaciens* transcriptome re-annotation.

### 4.6. RNAseq Expression Analysis

Bowtie 2 (v2.2.6) [86] was used to align the read1 to *S. ambofaciens* ATCC 23877 genome (GCF_001267885.1) as already described in Pinatel and Peano, 2018 [87]. For comparability reasons, ΩpirA rpoB(R) sequences were treated using the same pipeline and annotation previously available [34,35] and relative control stats are shown in Supplementary Table S1. Raw read counts relative to newly predicted ncRNAs were instead obtained for all three strains using FeatureCounts [88], and R package DESeq2 (v1.14.1) [89] was then used to produce differential expression data for each condition in biological duplicate, normalizing the counts to the total amount of reads mapped to the main chromosome in the less covered sample.

### 4.7. ncRNAs Annotation and Functional Predictions

The Rfam database (http://rfam.xfam.org/) was first used to search for functional information and the degree of phylogenetic conservation of predicted *S. ambofaciens* sRNAs. The Rfam database is a collection of RNA families (non-coding RNA genes, structured *cis*-regulatory elements, and self-splicing RNAs) by multiple sequence alignments, consensus secondary structures, and covariance models [90,91].

The degree of sRNAs conservation during microbial phylogeny was also analyzed by Global Automatic Small RNA Search go (GLASSgo) web server [46,47,48]. With the same method and tool, we performed a conservation analysis on ClassI asRNA. GlassGo was based on BLAST searches, pairwise identity filtering, and structure-based clustering. This tool does not allow predicting if ncRNA is expressed or not, however according to the data reported by the developers, the positive predictive value (PPV) is high (minimum of 0.85).

To predict the putative targets of each sRNA, we used Interacting RNAs (IntaRNA) web server [49,50]. This tool calculates the RNA-RNA interactions by an energy-based approach. IntaRNA predicts interacting regions between each sRNA and putative target mRNA by incorporating the accessibility of both interaction sites and the presence of a seed interaction; both features are commonly observed in sRNA–mRNA interactions [92].

COG functional classes were obtained from Conserved Domain Database (CDD) domain database https://www.ncbi.nlm.nih.gov/Structure/cdd/cdd.shtml (accessed on 18 June 2020) as reported by Tatà et al. [35]. Functional enrichment analysis was performed on class I asRNA by counting the total number of COG classes identified, considering the same class several times if the latter mapped on targets to which more than one class was assigned. Similarly, class II and III asRNAs (comprising two overlapping transcripts) were considered twice. The unclassified genes (without COG classification) were excluded from the analysis.

Codon usage in *Streptomyces coelicolor* was analyzed by the Codon Usage Database (https://www.kazusa.or.jp/codon/), accessed on 18 June 2020 [93], using codon count program (https://www.kazusa.or.jp/codon/countcodon.html), accessed on 18 June 2020.

### 4.8. Code Availability

#### 4.8.1. Gene Expression Analysis

The first read of each pair was aligned to *S. ambofaciens* genome (GCF_001267885.1_ASM126788v1_genomic.fna) according to the pipeline previously published [87]. The scripts are also available at https://github.com/epinatel/Bacterial_RNAseq/blob/master/RNAseq_analysis_pipeline.txt, accessed on 22 June 2021. All the tools’ versions correspond to those provided in the paper. Gene level counts obtained by the pipeline, adopting as reference *S. ambofaciens* annotation table published in [34] (datasheet 1), were normalized with factor sizes estimated by DESeq2 on the entire batch of data (3 genotypes and 4 timepoints). MasigPro analysis was run with default regression parameters (α = 0.05; Q = 0.05) and forward step regression model was adopted. Significant genes lists were obtained by imposing R-square = 0.7, in order to select genes correctly fitting the model as suggested by Conesa et al. (2006) [94].

#### 4.8.2. Transcriptome Re-definition

Rockhopper 2.0.3 [38,39,40] was run on *S. ambofaciens* wild-type raw read pairs separately for each timepoint. Predictions were merged and classified into UTR, ncRNA, and intercistronic regions, finally antisense transcription was identified using BEDTools functions [85]. 

#### 4.8.3. ncRNAs Differential Expression

The first read of each pair was aligned to *S. ambofaciens* genome as previously described for gene expression and FatureCounts (Subread v2.0) was adopted to obtain read counts based on the annotation reported in Supplementary Tables S4–S7. The following command line was used:

featureCounts -a <ncRNA.saf> -o <ncRNA_counts> -F SAF -t gene -s 1 -T 15 -p -B -C -J -G <GCF_001267885.1_ASM126788v1_genomic.fna> --fracOverlap 0.5 <BAM file list>

DESeq2 version 1.26.0 in R 3.6.3 with default parameters was run to obtain differentially expressed ncRNAs, manually defining the size factors according to the ones estimated on the entire transcriptome and already used for standard gene expression analysis.

## Figures and Tables

**Table 1:** Transcriptional unit classification in *S. ambofaciens* genome.

Transcriptional Units	Number	Length (Median)
Monocistronic	4433	902
Polycistronic	1154	2177
sRNA	45	100
asRNA I	119	166
**Transcriptional Units**	**Number**	**Overlap Length (Median)**
asRNA I	119	150
asRNA II	83	113
asRNA III (cutoRNA)	507	167

## Data Availability

Raw data are publicly available at Sequence Reads Archive under accession numbers: PRJNA342588 for the w.t. strain, PRJNA430852 for *pirA*::pTYM-18 (ΩpirA) and *pirA*::pTYM-rpoB(R) strains.

## Supplementary Materials

The following are available online at https://www.mdpi.com/article/10.3390/antibiotics10080947/s1, Figure S1 ncRNA identified by analysis of 3′ or 5′ UTRs. Figure S2. Codon frequencies in *S. ambofaciens* ATCC 23877 CDSs. Table S1. List of monocistronic and polycistronic transcriptional units identified in this study. Table S2. Re-defined transcriptome mapping statistics. Table S3. List of predicted sRNA. Table S4. List of predicted Class I asRNA. Table S5. List of predicted Class II asRNA. Table S6. List of predicted Class III asRNA. Table S7. Differential expression (log2Fold Change) of sRNA and Class I asRNA in w.t. strain at different growth time points (48, 72, 96 and 120 h). Table S8. Rfam predictions. Table S9. GlassGO conservation analysis of *S. ambofaciens* sRNAs. Table S10. IntaRNA target predictions of *S. ambofaciens* sRNAs. Table S11. GlassGO conservation analysis of *S. ambofaciens* Class I asRNA. Table S12. MaSigPro analysis of gene expression. Table S13. Differential expression (log2Fold Change) of Class I asRNA obtained comparing w.t., ΩpirA and ΩpirA rpoB(R) strains, according the 4 time points (48, 72, 96 and 120 h) and ΩpirA, and ΩpirA rpoB(R) mapping statistics.
