# Supplementary material for: Interplay between Non-Coding RNA Transcription, Stringent/Relaxed Phenotype and Antibiotic Production in Streptomyces ambofaciens
Source: Antibiotics (Basel). 2021 Aug 5;10(8):947. doi: 10.3390/antibiotics10080947 (PMC8388888; doi:10.3390/antibiotics10080947)
Supplement: Supplementary file 1 [file antibiotics-10-00947-s001.zip › antibiotics-1289361 supp fig.pdf]

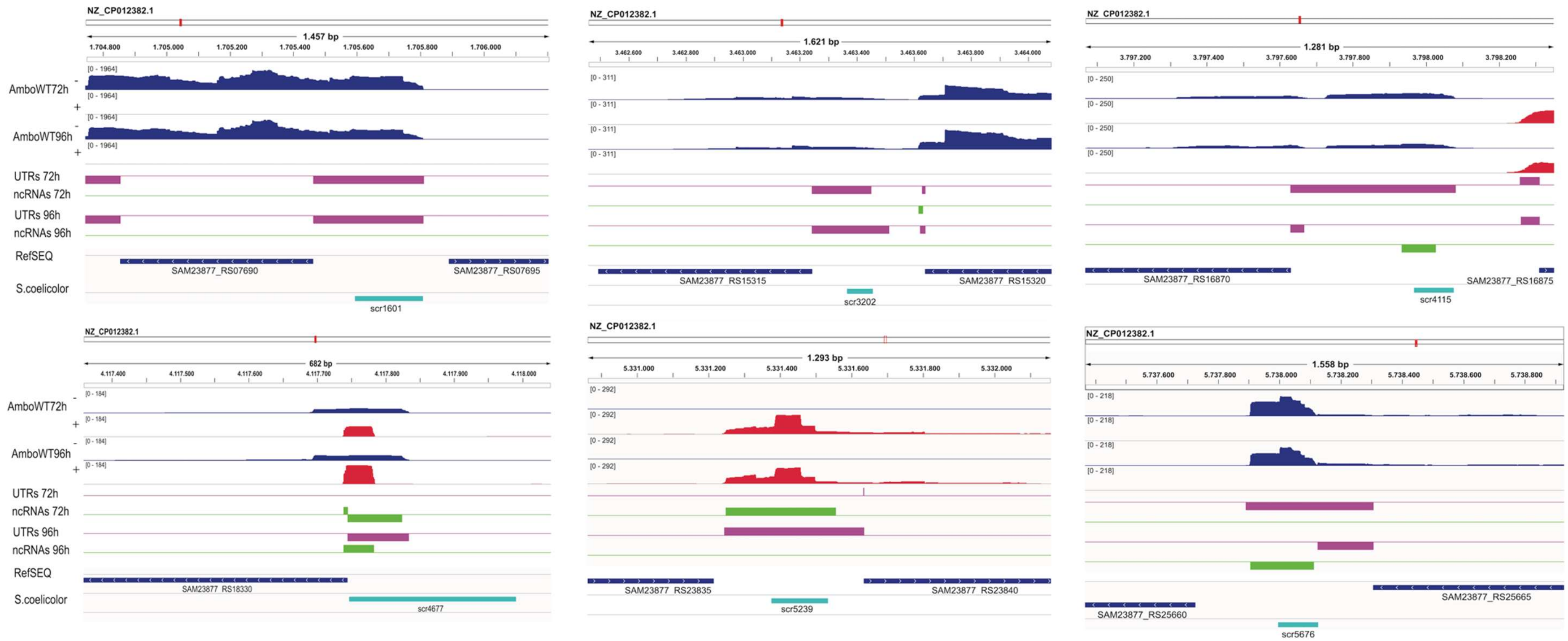

Figure S1. ncRNA identified by analysis of 3′ or 5′ UTRs.

										Bld					Whi				
					RS26275	RS31515	RS11875	RS29530	RS01800	RS07100	RS12125	RS23250	RS24355	RS25830	RS09475	RS14495	RS21730	RS25465	RS24125
	**description**	**Codon**	**as**	**SCO**	**RpoD**	**HrdC**	**HrdA**	**SigB**	**RpoE**	**BldD**	**bldM**	**BldKB**	**BldG**	**BldB**	**WhiA**	**WhiB**	**WhiD**	**WhiG**	**WhiE VII**
tRNA-Cys	Low frequency	UGU	-	0,07%	0,00%	0,00%	0,00%	0,00%	0,00%	0,00%	0,78%	0,00%	0,00%	0,00%	0,00%	0,00%	0,00%	0,00%	0,00%
tRNA-Cys	Common	UGC	asRNA #94 (RS28910)	0,72%	0,00%	0,00%	0,00%	0,75%	1,16%	0,00%	0,78%	0,85%	0,93%	0,00%	1,21%	4,55%	3,60%	0,00%	0,89%
tRNA-Gly	Low frequency	GGA	-	0,73%	0,20%	1,77%	0,00%	0,75%	0,58%	0,00%	1,17%	0,17%	2,78%	1,01%	0,30%	0,00%	1,80%	0,71%	0,00%
tRNA-Gly	Common	GGC	-	6,21%	3,72%	5,01%	4,38%	4,53%	3,47%	5,36%	2,72%	7,68%	9,26%	5,05%	6,06%	6,82%	4,50%	2,49%	4,46%
tRNA-Gly	Low frequency	GGU	-	0,94%	0,98%	0,29%	0,77%	0,00%	0,00%	2,38%	1,56%	1,02%	0,00%	1,01%	1,21%	0,00%	0,90%	0,71%	1,79%
tRNA-Gly	Common	GGG	asRNA #48 (RS18700)	1,85%	0,20%	0,00%	1,29%	0,38%	1,16%	0,60%	2,33%	0,51%	0,93%	1,01%	1,52%	0,00%	1,80%	3,20%	1,79%
tRNA-Leu	Low frequency	UUG	-	0,25%	0,00%	0,29%	0,00%	0,00%	1,16%	0,00%	0,78%	0,00%	0,00%	0,00%	0,30%	0,00%	0,00%	1,07%	0,00%
tRNA-Leu	Rare	CUA	-	0,04%	0,00%	0,00%	0,00%	0,38%	0,00%	0,00%	0,00%	0,00%	0,00%	0,00%	0,00%	0,00%	0,00%	0,00%	0,00%
tRNA-Leu	Rare	UUA	-	0,01%	0,00%	0,00%	0,00%	0,00%	0,00%	0,00%	0,00%	0,00%	0,00%	0,00%	0,00%	0,00%	0,00%	0,00%	0,00%
tRNA-Leu	Common	CUG	asRNA #94 (RS28915)	6,20%	4,31%	8,26%	6,44%	5,66%	6,94%	5,95%	3,11%	2,22%	12,04%	2,02%	6,97%	6,82%	4,50%	7,47%	5,36%
tRNA-Leu	Low frequency	CUU	-	0,16%	0,20%	0,29%	0,00%	0,38%	0,00%	0,60%	0,39%	0,00%	0,00%	1,01%	0,30%	0,00%	0,90%	0,00%	0,00%
tRNA-Leu	Common	CUC	-	3,72%	4,50%	2,65%	6,19%	3,40%	4,05%	5,36%	4,28%	2,73%	1,85%	5,05%	4,24%	4,55%	2,70%	2,85%	1,79%
tRNA-Met	Common	AUG	asRNA #63 (RS21105)	1,61%	1,76%	1,47%	0,77%	2,26%	2,31%	0,60%	3,89%	1,71%	2,78%	2,02%	1,52%	1,14%	2,70%	1,42%	2,68%
tRNA-Pro	Low frequency	CCA	asRNA #94 (RS28920)	0,14%	0,00%	0,00%	0,00%	0,00%	0,00%	0,00%	0,00%	0,00%	0,93%	0,00%	0,00%	0,00%	0,00%	0,36%	0,00%
tRNA-Pro	Common	CCG	-	3,40%	2,94%	1,77%	3,09%	2,26%	0,58%	3,57%	3,11%	3,07%	0,93%	3,03%	2,12%	0,00%	3,60%	1,78%	6,25%
tRNA-Pro	Common	CCC	-	2,59%	1,96%	2,95%	4,12%	3,02%	4,05%	1,79%	1,95%	1,54%	0,93%	1,01%	1,82%	2,27%	0,90%	2,85%	2,68%
tRNA-Pro	Low frequency	CCU	-	0,16%	0,00%	0,00%	0,00%	0,00%	0,00%	0,00%	0,78%	0,00%	0,00%	0,00%	0,00%	0,00%	0,00%	0,00%	0,00%

				Spiramycin							Antimycin		Pirin				
				RS26755	RS26760	RS26765	RS26770	RS26595	RS26680	RS26685	RS01785	RS01790	RS05960	RS11555	RS18305	RS31570	RS18295
	**description**	**Codon**	**as**	**DltE**	**PKS**	**PKS**	**PKS**	**smrS**	**smrB**	**smrR**	**KAS**	**NRPS**	**Pirin**	**Pirin**	**Pirin**	**Pirin**	**vLCAD-AcdB**
tRNA-Cys	Low frequency	UGU	-	0,09%	0,00%	0,19%	0,16%	0,26%	0,18%	0,15%	0,16%	0,00%	0,00%	0,46%	0,00%	0,00%	0,00%
tRNA-Cys	Common	UGC	asRNA #94 (RS28910)	0,18%	0,56%	0,51%	0,58%	0,26%	0,18%	1,07%	0,78%	0,63%	0,61%	0,46%	0,00%	0,93%	0,16%
tRNA-Gly	Low frequency	GGA	-	1,81%	1,05%	0,70%	0,79%	0,77%	0,73%	0,76%	0,70%	0,63%	1,52%	0,46%	1,23%	0,62%	0,00%
tRNA-Gly	Common	GGC	-	4,61%	7,08%	5,50%	4,93%	5,41%	6,90%	4,88%	7,11%	6,26%	6,97%	7,76%	7,98%	7,76%	7,88%
tRNA-Gly	Low frequency	GGU	-	1,90%	0,81%	2,72%	2,91%	1,80%	1,27%	0,76%	0,39%	0,38%	0,30%	0,91%	1,53%	0,93%	0,66%
tRNA-Gly	Common	GGG	asRNA #48 (RS18700)	1,90%	1,33%	3,42%	3,39%	2,84%	0,91%	2,13%	1,17%	0,97%	2,12%	2,28%	2,15%	1,24%	0,33%
tRNA-Leu	Low frequency	UUG	-	0,18%	0,07%	1,39%	1,69%	1,03%	0,73%	0,00%	0,23%	0,24%	0,00%	0,00%	0,31%	0,00%	0,00%
tRNA-Leu	Rare	CUA	-	0,00%	0,00%	0,00%	0,00%	0,00%	0,18%	0,00%	0,00%	0,03%	0,00%	0,00%	0,00%	0,31%	0,00%
tRNA-Leu	Rare	UUA	-	0,09%	0,00%	0,00%	0,00%	0,52%	0,00%	0,15%	0,00%	0,00%	0,00%	0,00%	0,00%	0,00%	0,00%
tRNA-Leu	Common	CUG	asRNA #94 (RS28915)	6,87%	7,54%	5,95%	5,40%	6,70%	7,80%	8,23%	5,31%	7,69%	5,45%	4,57%	4,60%	7,14%	5,09%
tRNA-Leu	Low frequency	CUU	-	0,09%	0,07%	0,00%	0,11%	0,26%	0,00%	0,15%	0,16%	0,03%	0,30%	0,00%	0,00%	0,00%	0,00%
tRNA-Leu	Common	CUC	-	5,24%	4,28%	2,59%	3,02%	3,61%	4,54%	4,73%	4,84%	4,18%	3,03%	2,28%	3,07%	4,97%	4,60%
tRNA-Met	Common	AUG	asRNA #63 (RS21105)	0,18%	1,09%	1,14%	1,11%	1,80%	1,45%	1,52%	0,86%	0,84%	1,82%	1,83%	4,29%	2,80%	2,79%
tRNA-Pro	Low frequency	CCA	asRNA #94 (RS28920)	0,09%	0,04%	0,06%	0,00%	0,26%	0,00%	0,15%	0,16%	0,07%	0,30%	0,00%	0,00%	0,00%	0,00%
tRNA-Pro	Common	CCG	-	2,71%	3,22%	2,78%	2,28%	1,55%	2,18%	2,29%	2,89%	2,75%	5,15%	2,74%	4,29%	4,35%	2,63%
tRNA-Pro	Common	CCC	-	4,52%	4,00%	3,54%	3,55%	3,61%	1,63%	3,20%	5,16%	4,42%	4,55%	3,65%	3,68%	3,73%	1,15%
tRNA-Pro	Low frequency	CCU	-	0,27%	0,04%	0,06%	0,16%	0,00%	0,00%	0,00%	0,08%	0,00%	0,00%	0,00%	0,31%	0,31%	0,00%

Figure S2. Codon frequencies in *S. ambofaciens* ATCC 23877 CDSs.
